# SIRT1-PGC1α-NFκB Pathway of Oxidative and Inflammatory Stress during *Trypanosoma cruzi* Infection: Benefits of SIRT1-Targeted Therapy in Improving Heart Function in Chagas Disease

**DOI:** 10.1371/journal.ppat.1005954

**Published:** 2016-10-20

**Authors:** Xianxiu Wan, Jian-jun Wen, Sue-Jie Koo, Lisa Yi Liang, Nisha Jain Garg

**Affiliations:** 1 Department of Microbiology and Immunology, University of Texas Medical Branch (UTMB), Galveston, Texas; 2 Department of Pathology, UTMB, Galveston, Texas; 3 Institute for Human Infections and Immunity, UTMB, Galveston, Texas; Universidade Federal do Rio de Janeiro, BRAZIL

## Abstract

Chronic chagasic cardiomyopathy (CCM) is presented by increased oxidative/inflammatory stress and decreased mitochondrial bioenergetics. SIRT1 senses the redox changes and integrates mitochondrial metabolism and inflammation; and SIRT1 deficiency may be a major determinant in CCM. To test this, C57BL/6 mice were infected with *Trypanosoma cruzi* (*Tc*), treated with SIRT1 agonists (resveratrol or SRT1720), and monitored during chronic phase (~150 days post-infection). Resveratrol treatment was partially beneficial in controlling the pathologic processes in Chagas disease. The 3-weeks SRT1720 therapy provided significant benefits in restoring the left ventricular (LV) function (stroke volume, cardiac output, ejection fraction etc.) in chagasic mice, though cardiac hypertrophy presented by increased thickness of the interventricular septum and LV posterior wall, increased LV mass, and disproportionate synthesis of collagens was not controlled. SRT1720 treatment preserved the myocardial SIRT1 activity and PGC1α deacetylation (active-form) that were decreased by 53% and 9-fold respectively, in chagasic mice. Yet, SIRT1/PGC1α-dependent mitochondrial biogenesis (i.e., mitochondrial DNA content, and expression of subunits of the respiratory complexes and mtDNA replication machinery) was not improved in chronically-infected/SRT1720-treated mice. Instead, SRT1720 therapy resulted in 2-10-fold inhibition of *Tc*-induced oxidative (H_2_O_2_ and advanced oxidation protein products), nitrosative (inducible nitric oxide synthase, 4-hydroxynonenal, 3-nitrotyrosine), and inflammatory (IFNγ, IL1β, IL6 and TNFα) stress and inflammatory infiltrate in chagasic myocardium. These benefits were delivered through SIRT1-dependent inhibition of NFκB transcriptional activity. We conclude that *Tc* inhibition of SIRT1/PGC1α activity was not a key mechanism in mitochondrial biogenesis defects during Chagas disease. SRT1720-dependent SIRT1 activation led to suppression of NFκB transcriptional activity, and subsequently, oxidative/nitrosative and inflammatory pathology were subdued, and antioxidant status and LV function were enhanced in chronic chagasic cardiomyopathy.

## Author Summary

In this study, we determined whether enhancing the activity of sirtuin 1 (SIRT1) would be beneficial in maintaining heart health in Chagas disease. SIRT1 senses the redox shifts and integrates mitochondrial metabolism and inflammation. We found that treatment with SIRT1 agonists, given in a therapeutic window of time after *Trypanosoma cruzi* infection, had no beneficial effects in reducing the cardiac remodeling and mitochondrial biogenic defects in chagasic mice. SIRT1 agonist, however, controlled the NFκB signaling of oxidative and inflammatory responses and helped preserve the left ventricular function in chagasic mice. Co-delivery of SIRT1 agonists with other small molecules that inhibit mitochondrial dysfunction, cardiac fibrosis, and parasite persistence will potentially form a complete therapeutic regimen against Chagas disease.

## Introduction

*Trypanosoma cruzi* (*T*. *cruzi* or *Tc*) is the etiological agent of Chagas disease that is endemic in Latin America [1]. After an exposure to parasite, infected individuals develop mild-to-no overt clinical symptoms. However, several decades later, ~30% of the infected individuals progress to heart failure associated with cardiac fibrosis, ventricular dilation, and thrombosis [2,3]. Vectors infected with *T*. *cruzi* are also present in the southern US [4], and CDC estimates that >300,000 infected individuals are living in the US [5,6]. Currently only two drugs are available for the treatment of *T*. *cruzi* infection: nifurtimox and benznidazole. These drugs are curative in early infection phase, but exhibit high toxicity and limited-to-no efficacy against chronic infection [7]. Thus, there is a need for new drugs for the treatment of chronic Chagas disease.

Mitochondria are the prime source of energy, providing ATP through oxidative phosphorylation (OXPHOS) pathway. A high copy number of mitochondrial DNA (mtDNA), reported to be ~6500 copies per diploid genome in myocardium [8], as well as the integrity of each mtDNA molecule is required to meet the high energy demand of the heart [9]. The mtDNA encodes 13 proteins that are essential for the normal assembly and function of the respiratory chain complexes. Peroxisome proliferator-activated receptor gamma coactivator-1α (PGC1α) is a member of the PGC family of transcription coactivators. PGC1α plays an important role in the expression of nuclear DNA and mtDNA encoded genes that drive mitochondrial biogenesis and increase the oxidative phosphorylation (OXPHOS) capacity [10]. Recently, we showed the mitochondrial respiratory chain activity and oxidative phosphorylation capacity were compromised in the myocardium of chronically infected rodents [11]. Further, mtDNA content and mtDNA encoded gene expression were decreased in *Tc*-infected cardiac myocytes and cardiac biopsies of chagasic patients [12]. Whether PGC1α activation of mitochondrial biogenesis and oxidative metabolism is compromised in CCM is not known.

Besides mitochondrial metabolic defects, chronic oxidative and inflammatory stress are hallmarks of Chagas disease. Acute infection by *T*. *cruzi* results in intense inflammatory activation of macrophages and CD8^+^T lymphocytes accompanied by increased expression of inflammatory mediators such as cytokines, chemokines, and nitric oxide synthase (NOS) in the heart (reviewed in [13,14]). Further, reactive oxygen species (ROS) are produced by neutrophils and macrophages activated by *T*. *cruzi* infection [14]. Besides infiltration of inflammatory infiltrate, cardiomyocytes are also reported to produce cytokines and mitochondrial ROS in response to *T*. *cruzi* infection [15,16]. The ROS induced adducts of DNA, protein and lipids were exacerbated in the myocardium of chronically infected rodents and human patients [12,17]. NFκB transcriptional factor signals oxidative and inflammatory responses [18], though mechanistic role of NFκB in chronic oxidative and inflammatory stress during CCM is yet to be elucidated.

Sirtuin 1 (SIRT1) is a highly conserved member of the family of NAD^+^-dependent Sir2 histone deacetylases, which deacetylates PGC1α at multiple lysine sites, consequently increasing PGC1α activity [19]. SIRT1 has also been reported to sense the redox shifts and integrate mitochondrial metabolism and inflammation through post-transcriptional regulation of the transcription factors and histones [20]. Several small molecule agonists of SIRT1 have been reported in literature. For example, resveratrol (3,5,4'-trihydroxy-trans-stilbene), a polyphenol found in red grape skins and red wine, is a natural agonist of SIRT1, and has been shown to increase mitochondrial number and the expression of genes for oxidative phosphorylation [21]. SRT1720 is a selective small molecule activator of SIRT1 and it is 1,000-fold more potent than resveratrol [22]. SRT1720 has been demonstrated to improve mitochondrial oxidative metabolism [23], and attenuate aging-related cardiac myocyte dysfunction [24].

In this study, we aimed to determine whether treatment with SIRT1 agonists will be beneficial in improving the heart function in Chagas disease. C57BL/6 mice were infected with *T*. *cruzi*, and treated with small molecule agonists of SIRT1. We demonstrate the therapeutic window of the efficacy of SIRT1 agonists in arresting the cardiac dysfunction in chagasic mice. Our results demonstrate a link between SIRT1, PGC1α, and NFκB in regulating ROS and inflammatory responses during *T*. *cruzi* infection and CCM.

## Results

**Fig 1:**
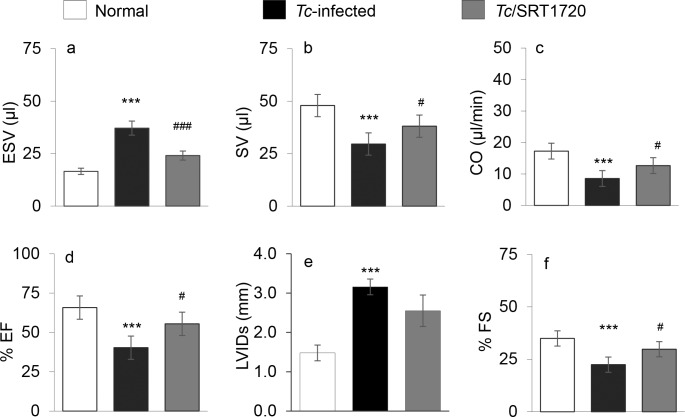
SRT1720 treatment improved the left ventricular function in chagasic mice. C57BL/6 mice were infected with *Trypanosoma cruzi* (10,000 *Tc*/mouse, ip), and treated with SRT1720 (SIRT1 agonist) during 45–66 days post-infection (pi), as described in Materials and Methods. Transthoracic echocardiography was performed by using a Vevo 2100 System to assess the left ventricular (LV) function at ~150 days pi. Shown are LV (a) end systolic volume (ESV), (b) stroke volume (SV), (c) cardiac output (CO), (d) ejection fraction (EF), (e) LV internal diameter at systole (LVIDs), and (f) fractional shortening (FS). Detailed data are presented in Table 1. For all figures, bar graphs show mean ± SD (n = 6–10 mice per group per experiment, triplicate observations per mouse per treatment, two experiments per group). Significance (*normal control vs. *Tc*-infected, ^#^ *Tc*-infected vs. *Tc*-infected/SRT1720-treated) was calculated by one-way ANOVA with Tukey’s test and shown as *^,#^p<0.05, **^,##^p<0.01, ***^,###^p<0.001.

We first determined if enhancing the SIRT1 activity would preserve the cardiac function in Chagas disease. Mice were infected with *T*. *cruzi* and then treated with resveratrol or SRT1720 as described in Materials and Methods. *In vivo* transthoracic echocardiography was performed at ~150 days pi to evaluate the changes in LV function (Table 1, Fig 1). Chagasic mice, as compared to normal controls, exhibited a substantial increase in LV end systolic volume (ESV, >2-fold), and a decline in stroke volume (SV, 35%), cardiac output (CO, 59%), and ejection fraction (EF, 33%) (Fig 1A–1D, all, p<0.001). The LV internal diameter at systole (LVID_s_) was increased by >2-fold, and consequently, fractional shortening (FS) was decreased by 53% in chagasic (vs. normal) mice (Fig 1E & 1F, p<0.001). The *Tc*-infected/SRT1720-treated mice, in comparison to normal controls, exhibited only 20%, 27% and 16% decline in SV, CO, EF, respectively, that were not statistically significant. In comparison to *Tc*-infected/untreated mice, infected/SRT1720-treated mice exhibited 55% decline in ESV and 29%, 33%, 37%, and 36% increase in SV, CO, EF, and FS, respectively (Fig 1Aa–1Ae, Table 1, all, ^#^p<0.05–0.001). *Tc*-infected/resveratrol-treated mice (vs. infected/untreated mice) exhibited a moderate (up to 20%) but significant improvement in *Tc*-induced loss in ESV, SV and CO; and a modest, but statistically insignificant, improvement in EF and FS (S1 Fig, panels a-f). These results suggested that SRT1720 treatment was effective in arresting the LV dysfunction in chagasic mice. Resveratrol provided a partial recovery of cardiac output in *Tc*-infected mice.

**Table 1:** Transthoracic echocardiography in *T*. *cruzi*-infected (± resveratrol or SRT1720 treatment) mice

Parameters	Mode	WT	WT x *T*. *cruzi*	^a^Resveratrol x	^b^SRT1720 x
					*T*. *cruzi*
				*T*. *cruzi*	
Heart rate (HR, beats per minute)	B mode	472 ± 51	464 ± 44	523± 63	489± 30
End systolic volume (ESV, μl)	B mode	16.56± 3.93	37.16± 2.82***	24.14± 2.95^###^	24.07± 0.83^###^
End diastolic volume (EDV, μl)	B mode	55.22± 7.72	51.42± 6.88	53.82± 7.26	58.65± 2.94
Stroke volume (SV = EDV–ESV, μl)	M mode	47.96± 2.08	29.60± 3.72***	39.88± 4.29^#^	38.11± 3.47^#^
Cardiac output (HR x SV, ml/min)	M mode	17.25± 2.76	8.52± 2.75***	15.60± 3.83^#^	12.66± 0.41^#^
% Ejection fraction (% EF = EDV-ESV x 100 / EDV)	M mode	65.78± 6.31	41.50± 5.60***	49.90± 8.47	55.48± 4.46^#^
LVID, end systole (LVID_s_, mm)	M mode	1.48± 0.21	3.16 ± 0.21***	3.18± 0.62	2.55± 0.66
% Fractional shortening (% FS = (LVIDd−LVID_s_) x 100 / LVID_d_	M mode	34.95± 5.24	22.44± 1.46***	21.41± 3.29	24.13 ± 2.67^#^
Interventricular septum (IVS_s_, mm)	M mode	0.84 ± 0.23	1.21 ± 0.22*	0.82 ± 0.16	1.25 ± 0.21
IVS_d_, mm	M mode	0.62± 0.14	1.04±0.20***	0.89± 0.13	0.79±0.19
LV posterior wall (LVPW_s_, mm)	M mode	1.45±0.30	0.85± 0.14***	0.97± 0.22	1.18± 0.21
LVPWd, mm	M mode	0.92± 0.17	0.67± 0.13*	0.86± 0.09	0.78± 0.23
IVS_s_ / LVPW_s_ ratio	M mode	0.59± 0.15	1.4± 0.37***	1.21± 0.25	0.94± 0.14
Area systole (mm^2^)	B mode	11.29± 2.67	15.77± 3.29*	10.48± 1.07	10.38± 0.67^#^
Area diastole (mm^2^)	B mode	17.67± 2.72	24.07±5.88*	16.36± 0.89	17.02± 1.46
LV mass (mg)	M mode	69.91± 10.44	97.86± 11.54***	73.53± 4.35	91.66± 3.04

C57BL/6 mice were infected with *T*. *cruzi* (10,000 parasites per mouse).

^a^ Mice were treated with resveratrol (20 mg/L) in drinking water during days 90-111post-infection (pi).

^b^ Mice were treated with SRT1720 (1 mg/mouse, intraperitoneal) during days 45–66 pi. Treatment was given three times a week.

Transthoracic echocardiography was performed in B and M mode at ~150 days post-infection using a Vevo 2100 System.

Data are presented as mean value ± SD. Significance is plotted as *normal vs. infected and ^#^WT.*Tc* vs. WT.*Tc* + treatment, and presented as *^,#^ <0.05, **^,##^ p<0.01, ***^,###^ p<0.001 (n = 6–10 per group per experiment).

**Fig 2:**
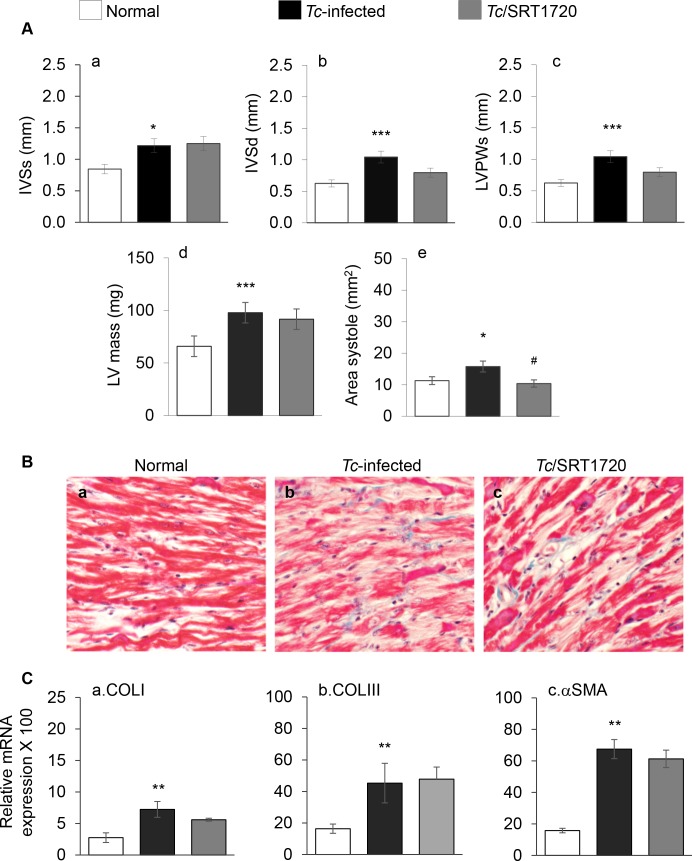
Effects of SRT1720 on cardiac remodeling in chagasic disease. Mice were infected with *T*. *cruzi*, treated with SRT1720, and examined at 150 days pi. **(A)** Cardiac structural changes were analyzed by echocardiography using a Vevo 2100 System. Shown are systolic (-s) and diastolic (-d) values for inter-ventricular septum (IVS) thickness (a&b), LV posterior wall (LVPW) thickness (c), LV mass (d), and LV area (e) in chronically infected mice. **(B)** Representative images show Masson's Trichrome staining of the heart tissue sections from (a) normal control, (b) *Tc*-infected, and (c) infected/ SRT1720-treated mice. **(C)** Quantitative real-time RT-PCR for collagen isoforms (COLI and COLIII, panel a&b) and alpha smooth muscle actin (αSMA, panel c). Data were normalized to GAPDH mRNA. Significance was calculated and presented as in Fig 1.

Echocardiography imaging in M mode was performed to gain an anatomo-pathological view of the heart in chronically infected mice. These data showed that systolic and diastolic thickness of inter-ventricular septum (IVS), LV area, and LV mass were increased by 44%, 40%, 28%, and 28% respectively, while LV posterior wall (LVPW) was thinned by 41% in chagasic (vs. normal) mice (Fig 2Aa–2Ae, all, p<0.001, Table 1). Histological evaluation of the tissue sections subjected to Masson's Trichrome staining showed an increase in diffused collagen deposition in chagasic myocardium (score: 4.0 ± 0.4 vs. 0.3 ± 0.04, *Tc*-infected vs. normal controls, p<0.05, Fig 2Ba & 2Bb). An increase in cardiac fibrosis in chagasic myocardium was also evidenced by 1.6-fold, 1.8-fold and 3.2-fold increase in mRNA levels for COLI, COLIII, and αSMA, respectively (Fig 2Ca–2Cc, all, p<0.01). In infected/SRT1720-treated mice, LV area (systole) was normalized (Fig 2Ae, ^#^p<0.05), though SRT1720 treatment provided no benefits in normalizing the IVS and LVPW thickness and LV mass in chagasic mice (Fig 2A). The myocardial deposition of collagen (score: 2.5 ± 0.8) and collagen-related gene expressions were also not significantly changed in SRT1720-treated (vs. untreated) chagasic mice (Fig 2B and 2C). Chagasic mice treated with resveratrol also exhibited modest control of LV mass, but no improvement in the IVS and LVPW thickness and LV area (S2 Fig, panels a-e, Table 1). Together, the results presented in Fig 2 and S2 Fig suggested that a) an increase in passive stiffness (enhanced ESV, IVS_s_, IVS_d_, LVID_s_) alongside thinning of the LVPW contributed to depressed LV function in chagasic mice, and b) SRT1720 benefits in arresting the LV dysfunction were not delivered through control of cardiac collagenosis and hypertrophy in chagasic mice.

**Fig 3:**
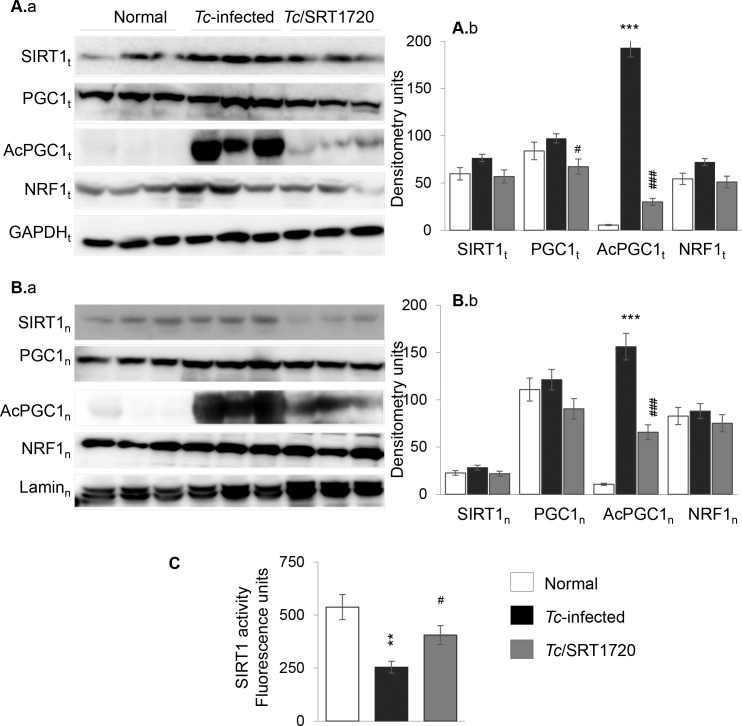
SIRT1 activity and nuclear localization of SIRT1, PGC1α, and NRF1 in chagasic mice (± SRT1720). Mice were infected with *T*. *cruzi*, treated with SRT1720, and harvested at ~150 days pi. Heart homogenates **(A)** and nuclear fractions **(B)** from normal and *Tc*-infected mice (±SRT1720 treatment) were submitted to Western blotting. Shown are representative immunoblots for myocardial (A.a) and nuclear (B.a) expression level of SIRT1, PGC1α (total and acetylated), and NRF1 in three mice per group. GAPDH and Lamin A/C were analyzed as controls. Densitometry analysis of bands from total homogenates (normalized to GAPDH) and nuclear fractions (normalized to Lamin A/C) was performed using the data from all mice in a group from two independent experiments and presented as mean value ± SD in A.b and B.b, respectively. **(C)** SIRT1 deacetylase activity in heart homogenates. Significance was calculated and presented as in Fig 1.

**Fig 4:**
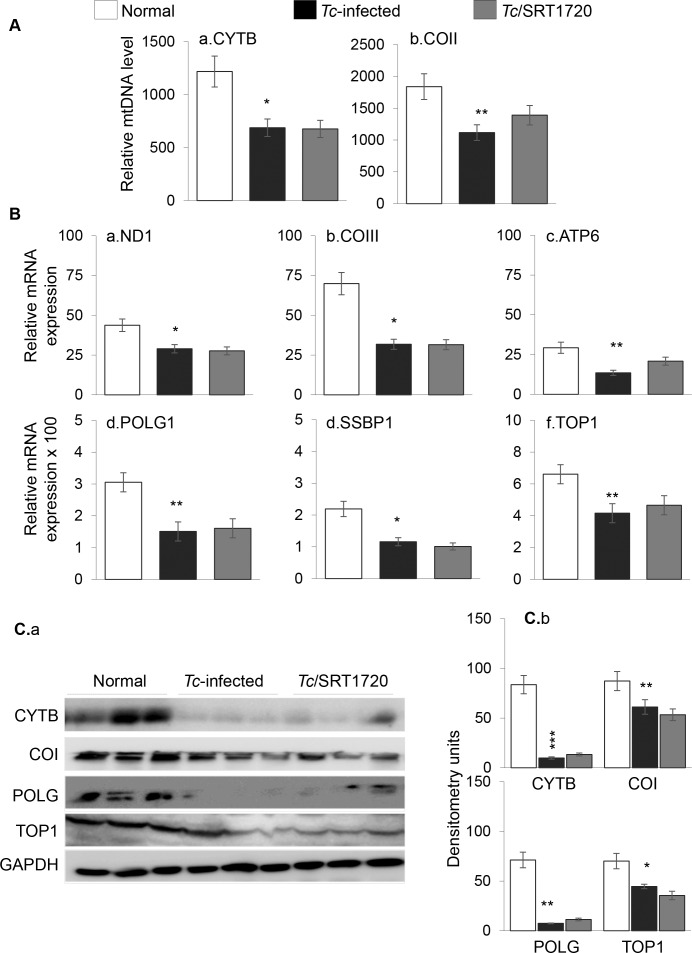
Mitochondrial biogenesis is compromised in chagasic mice (± SRT1720). C57BL/6 mice were infected with *T*. *cruzi*, treated with SRT1720, and harvested during chronic disease phase (150 days pi) **(A)** Myocardial mtDNA content was determined by real-time quantitative PCR amplification of CYTB (panel a) and COII (panel b) regions of mtDNA, and normalized to β-globin nuDNA. **(B)** Real-time quantitative RT-PCR for mtDNA-encoded transcripts (ND1, COIII, ATP6, a-c panels) and mtDNA replication/transcriptional machinery (POLG1, SSBP1, TOP1, d-f panels). For each target gene, *C*t values were normalized to GAPDH expression. **(C)** Shown are representative immunoblots for CYTB, COI, POLG, TOP1 and GAPDH (panel a). Densitometry analysis of the signal, normalized to GAPDH, is shown in panel b. Significance was calculated and presented as in Fig 1.

*T*. *cruzi* infection results in respiratory chain inefficiency in mice and humans [25,26]. SIRT1 deacetylates PGC1α, and PGC1α coactivation of nuclear respiratory factor (NRF1) signals the expression of key metabolic genes required for respiration and mtDNA transcription and replication. Western blotting showed the total and nuclear levels of SIRT1, PGC1α and NRF1 proteins were either increased or not changed in the myocardium of infected/untreated and infected/SRT1720-treated mice as compared to that noted in normal controls (Fig 3Aa, 3Ab and 3Ba, 3Bb). However, total and nuclear concentration of acetylated PGC1α (inactive form) were increased by >9-fold (Fig 3Aa, 3Ab and 3Ba, 3Bb, p<0.01), and associated with a 53% decline in SIRT1 activity (Fig 3C, p<0.05) in the myocardium of chronically infected (vs. normal) mice. SRT1720 treatment of chagasic mice resulted in 84% and 58% decline in total and nuclear levels of the acetylated PGC1α level, respectively (Fig 3A and 3B, ^#^p<0.001) and 60% increase in SIRT1 activity (Fig 3C, ^#^p<0.05). The changes in mitochondrial biogenesis were examined by measuring the mitochondrial markers at the DNA, gene expression and protein levels. The mtDNA levels for CYTB and COII sequences, normalized to nuclear DNA sequence for β-globin, were decreased by 25% and 24%, respectively, in the myocardium of chronically infected mice (Fig 4Aa & 4Ab, *p<0.05). No difference in the citrate synthase activity, a marker of mitochondrial mass, was noted in chagasic vs. normal mice. The mRNA levels for mtDNA encoded ND1, COIII, and ATP6 subunits that are essential components of the CI, CIV, and CV respiratory complexes and required for maintaining the oxygen consumption and coupled OXPHOS, were decreased by 34%, 55% and 54%, respectively, in chagasic (vs. normal) murine myocardium (Fig 4Ba–4Bc, *p<0.05). The protein levels of mtDNA-encoded CYTB and COI were also decreased by 60% and 33%, respectively, in chagasic myocardium (Fig 4Ca & 4Cb, *p<0.01). The decline in OXPHOS-related transcripts could be a result of changes in mtDNA replication/transcription efficiency. Our data showed the myocardial mRNA levels for mtDNA replication machinery, POLG1, SSBP1, and TOP1, were decreased by 51%, 47%, and 37%, respectively, in chagasic (vs. normal) mice (Fig 4Bd–4Bf, *p<0.05). We also noted 35%-90% decline in POLG and TOP1 protein levels in chagasic mice (Fig 4Ca & 4Cb, *p<0.01). Treatment with resveratrol resulted in 30–40% increase in mtDNA level (S3 Fig, panels A.a&b, ^#^p<0.05) and 40–55% increase in mRNA levels for mtDNA encoded ND1, COIII, and ATP6 genes (S3 Fig, panels B.a-c, ^#^p<0.05), and no significant improvement in the mRNA levels for POLG1, SSBP1 and TOP1 (S3 Fig, panels B.d-f) in chagasic mice. Surprisingly, SRT1720-treated/chagasic mice exhibited no significant improvement in the PGC1α/NRF1-dependent mtDNA content (COII and CYTB levels, Fig 4Aa & 4Ab), mtDNA encoded gene expression (ND1, COIII, ATP6, Fig 4Ba–4Bc), and mtDNA replication machinery (Fig 4Bd–4Bf) that were compromised in the myocardium of chagasic mice. Likewise, protein levels of the mtDNA-encoded proteins (e.g. CYTB, COI) and the mtDNA replication/transcription machinery (POLG1, TOP1) were not improved in SRT1720-treated chagasic mice (Fig 4Ca & 4Cb). Together, the results presented in Fig 3 and Fig 4 and S3 Fig suggested that a) mtDNA content and mRNA and protein levels of the mtDNA-encoded genes were significantly decreased in the myocardium of chronically infected mice, and this outcome was associated with a decline in mtDNA replication machinery, and b) SRT1720 treatment was effective in activation of SIRT1/PGC1α in the chagasic myocardium. However, c) SRT1720-mediated increase in SIRT1 activity and deacetylated PGC1α did not improve the mitochondrial biogenesis in chagasic mice.

**Fig 5:**
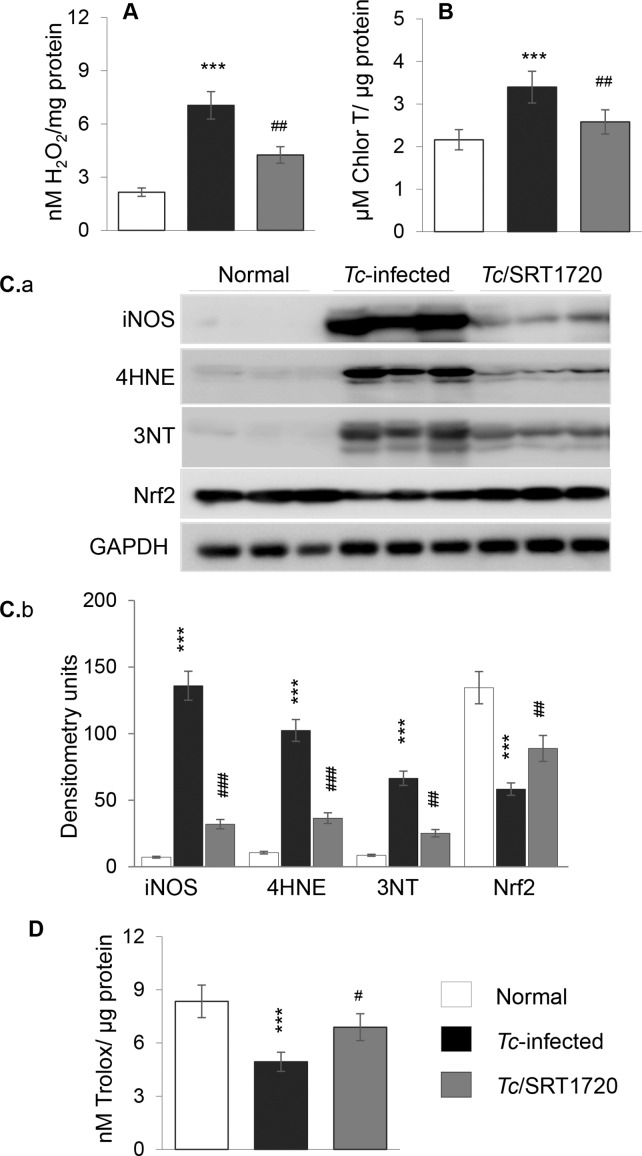
Oxidant/antioxidant status in chagasic mice (± SRT1720). Mice were *Tc-*infected, SRT1720-treated, and heart tissue harvested, as in Fig 4. Cardiac homogenates were used to measure **(A)** ROS (H_2_O_2_) and **(B)** advanced oxidation protein products (AOPP) levels. **(C)** Shown in panel a are representative immunoblots for inducible nitric oxide synthase (iNOS), 4-hydroxynonenal (4HNE), 3-nitrotyrosine (3NT), Nrf2, and GAPDH in cardiac homogenates of normal and *Tc*-infected (± SRT1720-treated) mice. Densitometry analysis of the western blot bands, normalized to GAPDH, is shown in panel b. **(D)** Total antioxidant capacity in cardiac homogenates. Significance was calculated and presented as in Fig 1.

We next determined if SIRT1 agonists controlled the chronic oxidative and inflammatory stresses that are hallmarks of Chagas disease [11]. Fluorometric evaluation of ROS showed 2.3-fold increase in H_2_O_2_ levels in the myocardial homogenates of chronically infected (vs. normal) mice (Fig 5A, *p<0.001). Advanced oxidation protein products (AOPPs) are formed by HOCl-induced chlorination of amines and considered a biomarker of inflammatory and oxidative pathology. Our data showed a 57% increase in AOPP content in chagasic (vs. normal) myocardium (Fig 5B, *p<0.01). The expression of inducible NOS (iNOS, a major source of nitric oxide) and the levels of the oxidative/nitrosative stress markers 4-hydroxynonenal (4HNE) and 3-nitrotyrosine (3NT) were increased by 20-fold, 10-fold, and 8-fold, respectively, in chagasic (vs. normal) myocardium (Fig 5Ca & 5Cb, all, p<0.001). In contrast, protein level of Nrf2 (transcriptional regulator of antioxidant gene expression) and total antioxidant capacity were decreased by 60% and 41%, respectively, in chagasic (vs. normal) myocardium (Fig 5C and 5D, *p<0.001). Resveratrol treatment was not effective in controlling the chronic oxidative stress in chagasic mice (S4 Fig, panels a&b). However, SRT1720 treatment resulted in a 57% and 90% decline in *Tc*-induced H_2_O_2_ and AOPP levels, respectively (Fig 5A and 5B, ^#^p<0.01); 76%, 64% and 62% decline in *Tc-*induced iNOS, 4HNE and 3NT levels, respectively (Fig 5Ca & 5Cb, all ^#^p<0.01); and 57% and 50% improvement in *Tc*-induced loss in Nrf2 expression and antioxidant capacity, respectively (Fig 5C and 5D, ^#^p<0.05). These results suggested that SRT1720 activation of the SIRT1 was beneficial in controlling the chronic oxidative stress in chagasic myocardium.

**Fig 6:**
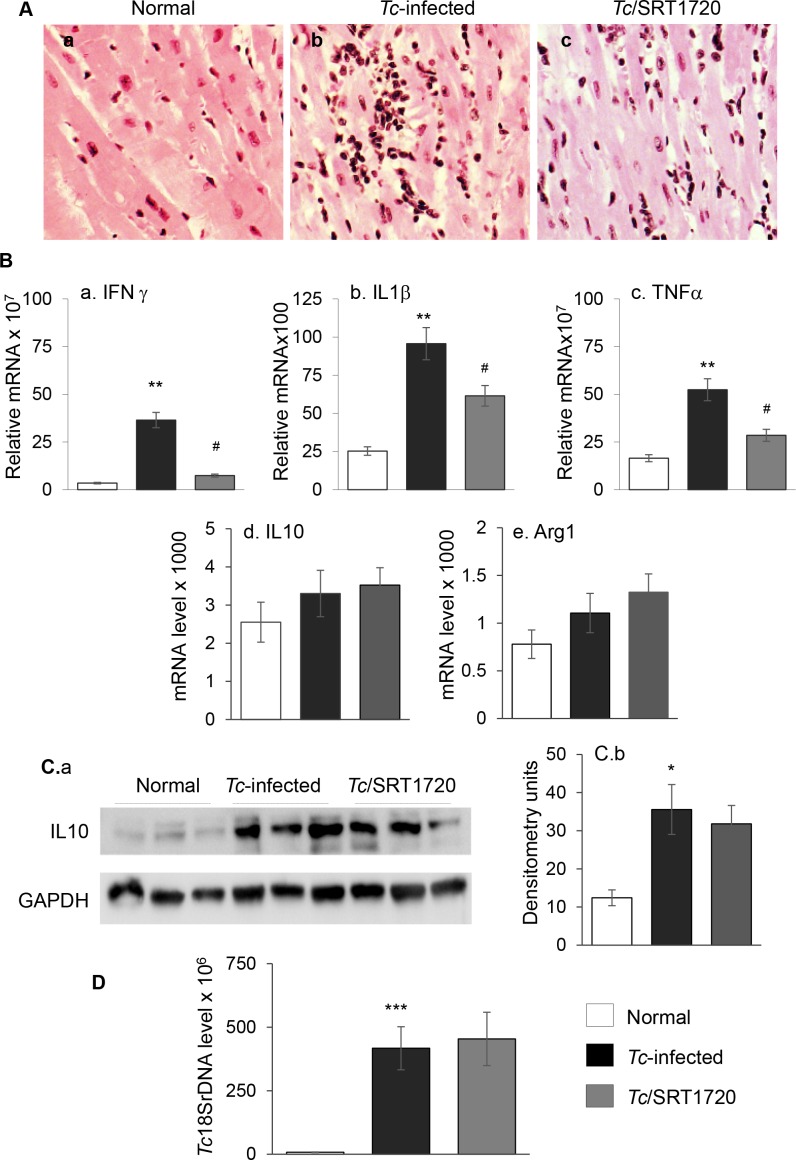
*T. cruzi* induced chronic inflammatory stress was controlled by SRT1720 treatment. Mice were *Tc-*infected, SRT1720-treated, and harvested at 150 days pi. **(A)** Hematoxylin and Eosin staining of LV tissue sections from (a) control, (b) *Tc*-infected, and (c) *Tc*-infected/SRT1720-treated mice are shown (pink: muscle/cytoplasm/keratin, dark brown: mononuclear infiltration). **(B)** Myocardial expression levels of (a) IFNγ, (b) IL1β, and (c) TNFα, (d) IL10 and (e) Arg1 were determined by real-time quantitative RT-PCR. For each target gene, *C*t values were normalized to GAPDH expression. **(C)** Shown in panel a are representative immunoblots for IL10 and GAPDH in cardiac homogenates of normal and *Tc*-infected (± SRT1720-treated) mice. Densitometry analysis of the western blot band for IL10, normalized to GAPDH, is shown in panel b. **(D)** Myocardial level of parasite burden was determined by quantitative PCR amplification of the *Tc*18SrDNA sequence. Significance was calculated and presented as in Fig 1.

Histological studies showed the myocardial level of inflammatory infiltrate constituted of diffused inflammatory foci (histological score: 2–3) was increased in heart tissue of chronically-infected/untreated (vs. normal) mice (Fig 6Aa & 6Ab). Chagasic mice exhibited a high degree of myocardial degeneration with enlarged myocytes. The cytokine gene expression was predominantly of proinflammatory nature evidenced by 9-fold, 3-fold and 2-fold increase in IFNγ, IL1β, and TNFα mRNA (Fig 6Ba–6Bc, all, *p<0.01) and 29% and 43% increase in IL10 and arginase 1 (Arg1) mRNA (Fig 6Bd & 6Be, *p<0.05), respectively, in chagasic myocardium. The myocardial IL10 protein level was increased by 3-fold in chagasic mice (Fig 6Ca & 6Cb, *p<0.05). Resveratrol treatment resulted in a modest (but not statistically significant) control of pro-inflammatory cytokine expression in chagasic heart (S5 Fig, panels a-c). However, myocardial inflammation was significantly subsided in infected/SRT1720-treated mice, evidenced by the detection of minimal tissue inflammatory infiltrate (histological score: 0–1, Fig 6Ac). Further, SRT1720-treated chagasic mice exhibited 80%, 36% and 46% decline in the expression of IFNγ, IL1β and TNFα, respectively (Fig 6Ba–6Bc, all, ^#^p<0.05), and no change in the myocardial expression of IL10 and Arg1 (Fig 6B & 6C). Chronic persistence of parasite was noted in all infected mice, and was not changed by SRT1720 treatment (Fig 6D). These results suggested that SRT1720 was beneficial in attenuating the myocardial inflammatory infiltrate and proinflammatory cytokine response in chagasic mice.

**Fig 7:**
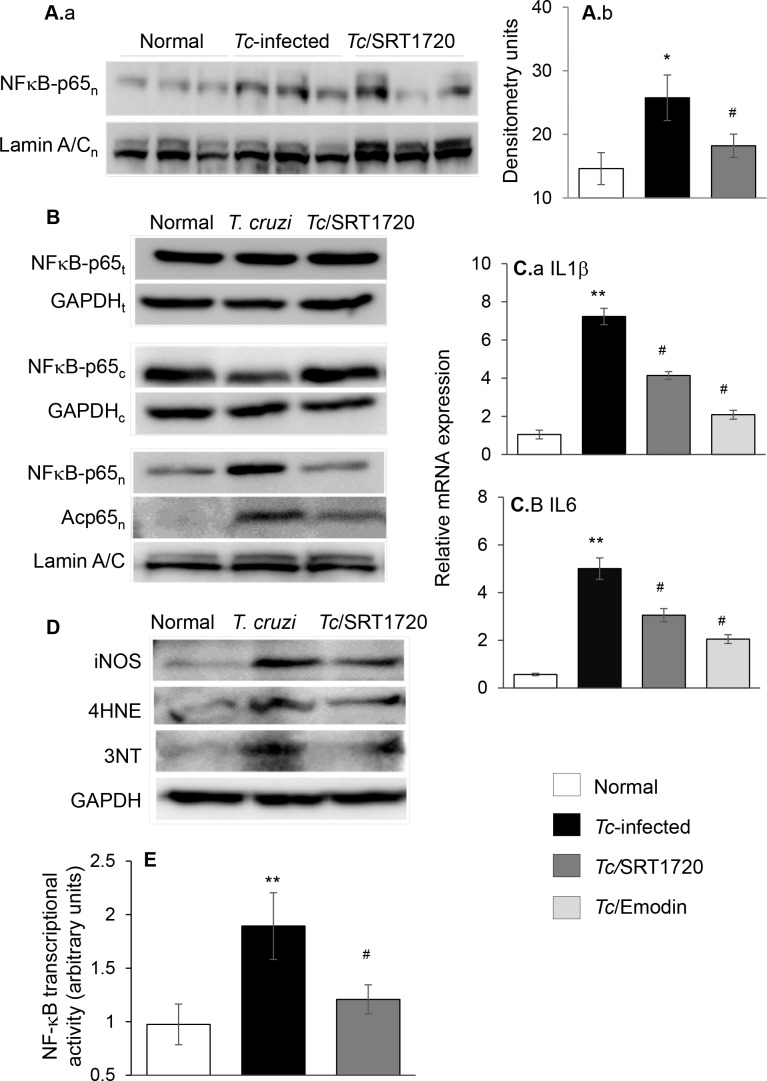
*T. cruzi* induced NF-κB transcriptional activity (± SRT1720). ***T*. (A)** Mice were *Tc-*infected, SRT1720-treated, and harvested at 150 days pi. Nuclear fractions from heart homogenates were prepared and submitted to Western blot analysis for NFκB p65 subunit and Lamin A/C (panel a). Densitometry analysis of NFκB p65 band, normalized to Lamin A/C, is shown in panel b. **(B)** Cardiac myocytes were infected with *T*. *cruzi* and incubated in presence or absence of 1 μM SRT1720 for 24 h. Total homogenates, and cytosolic and nuclear fractions were prepared as described in Materials and Methods. Shown are representative immunoblots for NFκB p65 subunit (total, cytosolic, and nuclear), and acetylated-p65 (nuclear) levels. Lamin A/C (nuclear fractions) and GAPDH (cytosolic and total homogenates) were analyzed for loading control. **(C)** Real time qRT-PCR measurement of mRNA levels for (a) IL1β and (b) IL6 in cardiomyocytes that were infected with *T*. *cruzi* and treated with SRT1720 (1 μM) or emodin (NFκB antagonist, 50 μM) for 24 h. Fold change was determined after normalizing the data with GAPDH mRNA. **(D)** Representative immunoblots for iNOS, 4-HNE and 3-NT (GAPDH control) levels in cell homogenates are shown. **(E)** NFκB transcriptional activity. HEK293 cells were transiently transfected with NFκB-TATA-luciferase reporter plasmid. Cells were co-transfected with a Renilla luciferase plasmid for normalization of transfection efficiency. Transfected cells were infected (cell: *Tc* ratio, 1:3) and incubated for 24 h in presence or absence of SRT1720. The relative NFκB transcriptional activity was measured by using a Dual Luciferase Assay System and normalized to Renilla luciferase activity. Bar graphs show mean value ± SD derived from triplicate experiments (3 replicates per group per experiment). Significance is shown as *^,#^p<0.05, **^,##^p<0.01, ***^,###^p<0.001 (*normal control vs. *Tc*-infected, ^#^ *Tc*-infected vs. *Tc*-infected and SRT1720- or emodin-treated).

NFκB family of transcription factors is of central importance in inflammation and immunity. Rel A (p65) is an important subunit of activated NFκB dimers (p50/p65, p65/p65, and p65/c-Rel). Western blotting showed the nuclear level of NFκB-p65 was increased by 76% in the myocardium of infected/untreated (Fig 7Aa & 7Ab, *p<0.05) mice, and normalized to control levels in infected/SRT1720-treated mice (^#^p<0.05), thus suggesting that SIRT1 might regulate NFκB activation in CCM. To verify this, we utilized an *in vitro* system. Cardiac myocytes were infected with *T*. *cruzi* and incubated for 24 h in presence or absence of SRT1720 or emodin (blocks IκB degradation and p65/Rel A release for nuclear translocation). As in chagasic heart, no changes in total levels of p65 were noted in any of the treatment groups, while the cytosolic level of p65 was decreased in *Tc*-infected cells (Fig 7B). Further, the nuclear translocation of p65 and acetylated-p65 were increased in *Tc*-infected cardiac myocytes (Fig 7B) and associated with 7-fold and 5-fold increase in the mRNA levels for IL1β and IL6, respectively (Fig 7Ca & 7Cb, *p<0.01). The *Tc-*induced cytokine gene expression was abolished by 59%-72% by emodin treatment (Fig 7Ca & 7Cb, ^#^p<0.05), thus, verifying the role of NFκB in signaling inflammatory responses in infected cardiomyocytes. SRT1720 treatment normalized the nuclear p65 content to control levels, substantially diminished the nuclear acetylated-p65 level (Fig 7B), and decreased the cytokine gene expression by 41%-43% (Fig 7C, ^#^p<0.05) in infected cardiomyocytes. SRT1720 treatment also decreased the *Tc*-induced oxidative stress (iNOS, 4HNE, 3NT) in cardiomyocytes (Fig 7D). We performed a dual reporter assay to evaluate the NFκB activity. HEK293 cells were transiently transfected with NFκB-TATA-luciferase reporter plasmid and pRL-TK plasmid (expresses renilla luciferase, control for transfection efficiency), infected with *T*. *cruzi* for 24 h, and NFκB-dependent luciferase activity was monitored. These data showed the NFκB-dependent luciferase activity (normalized to renilla luciferase) was increased by 2-fold in *Tc-*infected cells (Fig 7E, *p<0.01) and controlled by 66% when infected cells were treated with SRT1720 (Fig 7E, ^#^p<0.05). Together, these results suggested that SIRT1 deacetylation of NFκB-p65 regulated inflammatory responses that otherwise were pronounced in *T*. *cruzi*-infected cardiomyocytes.

## Discussion

In this study, we demonstrated that SIRT1 activity was decreased in chagasic heart, and treatment with SIRT1 agonist during a therapeutic window, i.e., after the immune control of acute parasitemia and before the onset of myocarditis, was beneficial in preserving cardiac function in CCM. The decline in SIRT1/PGC1α activity was not the key mechanism in mitochondrial biogenic defects in Chagas disease, and therefore SIRT1-targeted therapy did not normalize the PGC1α/NRF1-dependent mitochondrial biogenesis and cardiac remodeling in chagasic disease. Instead, SIRT1 deacetylation of NFκB-p65 repressed the *Tc*-induced inflammatory stress and preserved the antioxidant/oxidant balance in the myocardium of SRT1720-treated chagasic mice. Our results, to the best of our knowledge, provide the first evidence for potential utility of SRT1720 mediated protection of LV function in CCM.

Others and we have shown the mitochondrial respiratory complexes and OXPHOS capacity are compromised in the cardiac biopsies of *Tc-*infected experimental animals and chagasic human patients [12,17,27]. Our findings in this study suggested that a decline in mitochondrial biogenesis constituted at least one of the mechanisms involved in OXPHOS inefficiency in Chagas disease. This is because mtDNA content as well as the expression of the mtDNA encoded genes at mRNA and protein levels were significantly suppressed in chagasic myocardium (Fig 4); and mtDNA encoded 13 polypeptides are essential for normal assembly and function of the CI, CIII, CIV and CV complexes of the respiratory chain. The expression levels of SIRT1, PGC1α and NRF1 that are involved in regulating the mitochondrial biogenesis were not changed, yet SIRT1 activity and deacetylated-PGC1α (active form) were significantly decreased in chagasic heart (Fig 3). Though a decline in PGC1 isoforms (PGC1α and PGC1β) is noted in other metabolic diseases, such as obesity and diabetes [28–31]; it is generally accepted that deacetylation, and not the changes in the expression level, of PGC1α is required for maintaining the mitochondrial biogenesis.

We postulated that SIRT1 agonists, via enhancing the SIRT1/PGC1α activity, would offer a therapeutic option to improve the mitochondrial biogenesis, and subsequently, the heart function in CCM. We, first, used resveratrol as a therapeutic candidate for the treatment of chronic CCM. Resveratrol has been shown to induce mitochondrial biogenesis in many tissues [32,33], control pressure overload induced hypertrophy and contractile dysfunction in mice [34,35]; and reverse the ischemia/reperfusion induced loss in renal mitochondrial mass by an increase in the expression of PGC1α and its downstream mediators [36]. In the present study, though resveratrol partially improved the heart function (S1 Fig), an overall lackluster performance of resveratrol in arresting *Tc*-induced cardiac remodeling and mitochondrial biogenic defects was noted (S2–S5 Figs). This was despite the fact that we have used biologically relevant concentrations of resveratrol as was used in other studies. Our data suggest that delayed treatment in chronic phase when oxidative/inflammatory pathology have already caused tissue damage in the heart was at least partially responsible for resveratrol inefficacy in CCM. The data discussed below with SRT1720 treatment allow us to propose that SIRT1 agonists offered during the clinically asymptomatic phase when host has controlled the acute parasitemia but yet not entered the chronic phase of progressive cardiomyopathy, will be most beneficial in arresting the adverse clinical outcomes in Chagas disease.

We treated mice with SRT1720 (specific and potent SIRT1 agonist) for three weeks during 45–66 days pi. In contrast to untreated/infected mice that developed significant LV systolic dysfunction by ~150 days pi; short-term SRT1720 treatment in the clinically asymptomatic phase was effective in preserving the heart function in chronically infected mice (Fig 1). This is the first study demonstrating that SRT1720 treatment rescued the heart function following a chronic *T*. *cruzi* infection. The effects of SRT1720 in improving the LV function in chagasic mice were associated with a significant increase in SIRT1 activity and deacetylation of PGC1α (Fig 3), as has also been noted in models of metabolic disease [37,38]. Others have shown that long-term SRT1720 treatment produced benefits in increasing the organ function and lifespan in mice [24]. SRT1720 stimulated the mitochondrial biogenesis and effectively reversed the conditions associated with metabolic deficiencies [37,39,40]. Surprisingly, despite SIRT1 activation and PGC1α deacetylation suggested to be required for mitochondrial biogenesis, SRT1720 treatment did not reverse the mitochondrial biogenic defects in the myocardium of chagasic mice (Fig 4). A recent study showed the deacetylation by SIRT1 decreased PGC1α activity and mitochondria number in myotubes [41]. Others have shown that kidney-specific overexpression of SIRT1 was protective against metabolic kidney disease though mitochondrial number was not changed. Further studies will be required to delineate the SIRT1/PGC1α dynamics in maintaining the mitochondrial biogenesis in normal and disease conditions. Yet, our data allows us to surmise that activators of the sirtuin family of proteins may be important in the development of new therapeutic strategies for treating cardiac dysfunction in Chagas disease.

Multiple sources of ROS including mitochondrial electron transport chain leakage and NADPH oxidases, sometimes in response to cytokines and growth factors, are noted in Chagas disease (reviewed in [42]). In this study, we found that SIRT1 agonists enhanced the antioxidant capacity and reversed the oxidative/nitrosative injuries (Fig 5 & S4 Fig), inflammatory cytokine response (Fig 6 and Fig 7 and S5 Fig), and infiltration of inflammatory infiltrate in the myocardium of chronically infected mice (Fig 6). Consistent with these results, SRT1720 has been reported to decrease the levels of 3-nitrotyrosine and iNOS in ischemia perfusion induced renal injury in mice [36]. SRT1720 was also shown to ameliorate vascular endothelial dysfunction by enhancing COX2 signaling and reducing oxidative stress and inflammation with aging in mice [43]; and increase the levels of catalase, thus reducing ROS level and apoptosis and retaining kidney function in mice [44]. SIRT1 can deacetylate the FoxO factors and stimulate the expression of antioxidants [45], and inhibit NFκB signaling that is a major inducer of inflammatory responses [46]. The role of FoxO in preserving antioxidant/oxidant balance in CCM remains to be investigated. However, others and we have shown the activation of NFκB by *T*. *cruzi* in a variety of immune and non-immune cells [16,47]. A variable degree of loss in SIRT1 activity associated with steady hyper-activation of NFκB-p65 is observed in many chronic inflammatory diseases [48], including CCM in this study (Fig 7). SRT1720 treatment inhibited the nuclear translocation of p65/Rel A, NFκB transcriptional activity, and NFκB-dependent inflammatory cytokines’ gene expression in cells infected by *T*. *cruzi* (Fig 7). SIRT1 influenced the chronic inflammation in chagasic disease by directly deacetylating the p65/Rel A (Fig 7). SIRT1 can also inhibit the NκB target genes by co-localizing with p65 and p300, the latter a histone acetyl transferase with a broad range of substrates. While SIRT1’s ability to regulate NFκB activity is shown in macrophages [49,50], ours is the first observation demonstrating SIRT1 regulation of NFκB in stressed cardiomyocytes. The observation of no increase in *T*. *cruzi* burden in mice treated with SRT1720 (Fig 6) implies that NF-κB-induced inflammatory responses were more detrimental to the host than to the parasite. Further, our finding that SIRT1 agonist (SRT1720) restricted the ROS and oxidative stress markers (3NT and 4HNE) that otherwise were significantly induced by *T*. *cruzi* infection (Fig 6) suggest that SIRT1/NFκB axis coordinates the oxidative stress as well as inflammation in chronic CCM and heart failure.

In summary, we have shown that mitochondrial biogenesis is compromised in chronic chagasic mice. A loss of SIRT1 activity contributed to NFκB-p65 activation and chronic cardiac pathology and heart failure in CCM. SRT1720 treatment enhanced the SIRT1/PGC1α activity but failed to improve the mitochondrial biogenesis in CCM. Instead, SRT1720 influenced the SIRT1/NFκB regulation of oxidative, nitrosative, and inflammatory responses, and, consequently, preserved the cardiac function in chagasic mice. We conclude that activators of the sirtuin family of proteins will provide promising new therapeutic strategies for treating cardiac dysfunction in chronic Chagas disease.

## Materials and Methods

### Ethics statement

All animal experiments were performed according to the National Institutes of Health Guide for Care and Use of Experimental Animals, and approved by the Institutional Animal Care and Use Committee (IACUC) at the University of Texas Medical Branch, Galveston (protocol number: 0805029).

### Mice and parasites and cell culture

All chemicals were of molecular grade, and purchased from Sigma-Aldrich (St. Louis, MO) unless otherwise stated. *T*. *cruzi* trypomastigotes (SylvioX10 strain, ATCC 50823) were propagated by *in vitro* passage in C2C12 cells. C57BL/6 mice were purchased from Harlan Laboratories (Indianapolis, IN). Mice (6-weeks-old) were infected with *T*. *cruzi* (10,000 trypomastigotes/mouse, intraperitoneal), and harvested at days 150 post-infection (pi) corresponding to chronic disease phase. To enhance the SIRT1 activity, two approaches were applied. One, mice were treated with resveratrol (20 mg/ml in drinking water) for three weeks, during days 90–111 pi. Two, mice were given SRT1720 (1 mg/100 μl/mouse, intraperitoneally, Selleck Chemicals, Houston, TX) three times a week during days 45–66 pi. Tissue samples were stored at -80°C. Protein levels were determined by using the Bradford Protein Assay (Bio-Rad, Hercules CA).

Human cardiomyocytes were cultured and maintained in Dulbecco's modified Eagle's medium/F-12 medium with 12.5% fetal bovine serum. Cardiomyocytes were seeded in T75 flasks (3×10^6^ cells per flask, 70% confluence), and infected with *T*. *cruzi* trypomastigotes (cell: parasite ratio, 1:3). Cells were incubated in presence or absence of SIRT1 agonist (1 μM SRT1720) and NF-κB inhibitor (50 μM emodin) for 24 h.

### Echocardiography assessment of LV function

Mice were continuously anesthetized by inhalant 1.5% isoflurane/100% O_2_ to maintain a light sedation level. Mice were placed supine on an electrical heating pad at 37°C and heart rate and respiratory physiology were continuously monitored by electrocardiography. Mice chests were shaved, and warm ultrasound gel was applied to the area of interest. Transthoracic echocardiography was performed using the Vevo 2100 ultrasound system (Visual Sonics, Toronto, Canada) equipped with a high-frequency linear array transducer (MS400, 18–38 MHz) [51]. Heart was imaged in B-mode and M-mode to examine the parameters of left ventricle (LV) in diastole (-d) and systole (-s). All measurements were obtained in triplicate and acquired in long-axis and short-axis views. Data were analyzed by using Vevo 2100 standard measurement software.

### Histology

Histological preparation and staining of the tissues was performed at the Research Histopathology Core at the UTMB. Briefly, tissue sections were fixed in 10% buffered formalin, dehydrated in absolute ethanol, cleared in xylene, and embedded in paraffin. Five-micron tissue sections were subjected to Masson’s Trichrome or Hematoxylin and Eosin (H&E) staining, and evaluated by light microscopy using an Olympus BX-15 microscope equipped with a digital camera and Simple PCI software (v.6.0, Compix, Sewickley, PA). In general, we analyzed each tissue section for >10 microscopic fields (20X magnification), and examined three different tissue sections/mouse (4 mice/group). The collagen area as a percentage of the total myocardial area was assessed as a measure of fibrosis. All pixels with blue stain in Masson’s trichrome-stained sections were selected to build a binary image, subsequently calculating the total area occupied by connective tissue. Sections were categorized based on percent fibrotic area as: (0) <1%, (1) 1–5%, (2) 5–10%, (3) 10–15%, and (4) >15% [52].

Myocarditis (presence of inflammatory cells) was scored as 0 (absent), 1 (focal/mild, ≤1 foci), 2 (moderate, ≥2 inflammatory foci), 3 (extensive coalescing of inflammatory foci or disseminated inflammation), and 4 (diffused inflammation, tissue necrosis, interstitial edema, and loss of integrity). Inflammatory infiltrate was characterized as diffused or focal depending upon how closely the inflammatory cells were associated [52].

### Gene expression analysis

Heart tissue sections (10 mg) were homogenized in 500 μl of TRIzol reagent (Invitrogen, Carlsbad, CA), and RNA was extracted by chloroform/isopropanol/ethanol method. Total RNA (2 μg) was reverse transcribed by using poly(dT)18 with an iScript kit (Bio-Rad). The cDNA was utilized as template with SYBR-Green super-mix (Bio-Rad), and real time quantitative PCR (qPCR) was performed on an iCycler Thermal Cycler. The gene-specific oligonucleotide pairs used for amplifying the mRNAs are listed in supplemental S1 Table. The PCR Base Line Subtracted Curve Fit mode was applied for threshold cycle (Ct), and mRNA level was calculated by iCycler iQ Real-Time Detection Software (Bio-Rad). The Ct values for target mRNAs were normalized to geometric mean of GAPDH mRNA, and the relative expression level of each target gene was calculated as 2^−ΔCt^, where ΔC_t_ represents the C_t_ (sample)—C_t_ (control) [53,54].

### Tissue and cell homogenates and fractionation

Freshly harvested heart tissue sections (30 mg) were washed with ice-cold Tris-buffered saline and homogenized in RIPA buffer (tissue: buffer ratio, 1: 10, w/v) [17]. Homogenates were centrifuged for 10 min at 10,000 g, and supernatants were stored at −80°C. For the preparation of nuclear and cytosolic fractions, heart tissue sections (50 mg) were homogenized in ice-cold HMK buffer (10 mM HEPES pH 7.9, 1.5 mM MgCl_2_, 10 mM KCl) containing 1 mM DTT and 1% Protease Inhibitor Cocktail (Sigma-Aldrich). Tissue lysates were centrifuged at 4°C, 10000 g for 20 min and supernatants stored as a cytosolic fraction. The pellets were re-suspended in HMK buffer containing 0.42 M NaCl, 0.2 mM EDTA, and 25% (v/v) glycerol, centrifuged at 4°C, 20, 000 g for 5 minutes, and nuclear fractions were stored at −80°C.

Cardiomyocytes were infected with *T*. *cruzi* and incubated in presence or absence of SRT1720 (with 1 μM) for 24 h. Cells were lysed for 30 min on ice in lysis buffer containing 50 mM Tris pH 7.5, 150 mM NaCl, 1 mM EDTA, 1 mM EGTA, 1% NP-40, 2.5 mM KH_2_PO_4_, and 1 mM Na_3_VO_4_. Cell lysates were centrifuged at 3000 g at 4°C for 15 min and the resultant supernatants were stored at −80°C. For the preparation of nuclear and cytosolic fractions, cells (7×10^6^/ml) were incubated on ice for 30 minutes in buffer A (10 mM HEPES, pH 7.9, 10 mM NaCl, 0.1 mM EDTA, 0.1 mM EGTA, 1 mM DTT, 1 mM PMSF) containing 0.625% NP-40 and 1% protease inhibitor cocktail. Cell lysates were centrifuged at 4°C at 10,000 g for 1 min and supernatants stored as a cytosolic fraction. Pellets were washed with buffer A containing 1.7 M sucrose, re-suspended in buffer B (20 mM HEPES pH 7.9, 0.4 M NaCl, 1 mM EDTA, 1 mM EGTA, 1 mM DTT, and 1 mM PMSF), and centrifuged at 4°C at 13 000 g for 5 minutes. The resultant supernatants were stored at −80°C as nuclear extracts.

### Western blotting

Heart or cell homogenates and nuclear fractions (30 μg protein) were electrophoresed on a 4–15% Mini-Protein TGXTM gel using a Mini-PROTEAN electrophoresis chamber (Bio-Rad), and proteins were transferred to a PVDF membrane using a Criterion Trans-blot System (Bio-Rad). Membranes were blocked with 50 mM Tris, 150 mM NaCl (TBS) containing 5% non-fat dry milk (NFDM), washed three times for 10 min each with TBS—0.1% Tween 20 (TBST), and incubated overnight at 4°C with antibodies against CYTB (Santa Cruz Biotech, Dallas, TX, sc11436), COI (Abcam, Cambridge, UK, ab147053), GAPDH (Cell signaling, Danvers, MA, 3683), 4-hydroxynonenal (4HNE, Alpha Diagnostic Inc, San Antonio, TX, HNE11-S), iNOS (Abcam, ab49999), Lamin A/C (Santa Cruz, sc20681), NF-κB-p65 subunit (Santa Cruz, F-6 clone, sc-8008); NF-κB-acetyl- p65 (Abcam, acetyl K310 clone, ab198703), nitrotyrosine (3NT, Merck Millipore, Billerica, MA, 06–284), NRF1 (Santa Cruz, sc33771), Nrf2 (Santa Cruz, sc722), PGC1α (Santa Cruz, sc13067), POLG (Santa Cruz, 390634), SIRT1 (Abcam, ab32441), TOP1 (Abcam, ab3825), IL-10 (A2, Santa Cruz, sc-365858) and anti-acetylated-lysine antibody (Cell Signaling, 9441). All antibodies from Santa Cruz were used at 1:200 dilution in TBST-5% NFDM. All other antibodies were used at 1:1000 dilution in TBST-5% NFDM. Membranes were washed as above, incubated with HRP-conjugated secondary antibody (1:10,000 dilution, Southern Biotech, Birmingham, AL), and images were acquired by using an ImageQuant LAS4000 system (GE Healthcare, Pittsburgh, MA). Immunoblots were subjected to Ponceau S staining to confirm equal loading of samples. Densitometry analysis of protein bands of interest was performed using a Fluorchem HD2 Imaging System (Alpha-Innotech, San Leandro, CA), and normalized against GAPDH (tissue homogenates) or Lamin A/C (nuclear fractions).

### SIRT1 activity

SIRT1 deacetylase activity was measured by using a SIRT1 Fluorometric Assay Kit (Abcam, ab156065). Briefly, nuclear fractions (100 μg) isolated from heart tissues were added to acetylated Lys^382^ p53 peptide (50 μl) that is coupled with fluorophore and quencher at the amino terminal and carboxyl terminal, respectively. The reaction was initiated with addition of 100 μl NAD^+^, and SIRT1 dependent deacetylation of the substrate peptide coupled with its digestion by the action of proteases, and fluorescence release recorded. The fluorescence intensity (Ex_350nm_/Em_440nm_) was measured at two min intervals for 60 minutes using a SpectraMax M5 microplate reader (Molecular Devices, Sunnyvale CA). Standard curve was prepared with recombinant SIRT1 (0–120 ng), and results were presented as relative fluorescence units per μg protein.

### Mitochondrial DNA (mtDNA) and *T*. *cruzi* DNA (*Tc*DNA)

Tissue sections (5 mg) were subjected to Proteinase-K lysis, and total DNA was purified using a DNeasy Blood & Tissue Kit (Qiagen, Hilden, Germany). Total DNA (20 ng) was utilized as template with SYBR Green Super-mix (Bio-Rad) and primer pairs specific for mtDNA-encoded cytochrome b (CYTB) and cytochrome oxidase 2 (COII) regions, and real-time qPCR was performed on an iCycler thermal cycler. The mtDNA content was normalized to β-globin nuDNA. For the evaluation of parasite burden, *Tc*18SrDNA-specific primers were utilized for qPCR, and data were normalized to GAPDH.

Citrate synthase (CS) activity is a sensitive measure of mitochondrial mass, and was measured by using the MitoCheck Citrate Synthase Activity Assay Kit (Cayman, Ann Arbor, MI) following the protocol provided by the manufacturer.

### Oxidant and antioxidant levels

To measure the total H_2_O_2_ levels, tissue homogenates (100 μg) were added in triplicate to flat-bottom (dark-walled) 96-well plates. The reaction was started with addition of 33-μM amplex red (10-acetyl-3, 7-dihydroxyphenoxazine) and 0.1U/ml horseradish peroxidase (final reaction volume: 150 μl). The oxidation of amplex red to fluorescent resorufin by H_2_O_2_ (Ex_563nm_/Em_587nm_) was recorded on a SpectraMax M2 microplate reader (Molecular Devices). Standard curve was prepared with 0–5 μM H_2_O_2_.

Advanced oxidation Protein Products (AOPP) are produced through the reaction of proteins with chlorinated oxidants (e.g. chloramines, hypochlorous acid), and provide a sensitive measure of total oxidative stress. AOPP contents were assayed by using the OxiSelect AOPP Assay Kit (Cell Biolabs, San Diego, CA). Briefly, tissue homogenates (10 μg) were mixed with 10 μl of 1.16 M KI and 20 μl of 100% acetic acid (final volume: 200 μl). Reaction was stopped after 5 min and absorbance was recorded at 340 nm. AOPP concentration was expressed as chloramine-T equivalents (standard curve: 0–100 μmol chloramine-T/ml) [55].

A commercially available kit (Abcam ab65329) was utilized to measure the total antioxidant capacity (TAC). The assay uses lag time by antioxidants against the myoglobin-induced oxidation of 2,2'-azino-di(3-ethylbenzthiazoline-6-sulfonic acid (ABTS) with H_2_O_2_. Briefly, 20 μl of tissue homogenates (diluted 1:20, v/v) were added in triplicate to 96-well plates, and mixed with 90 μl of 10 mM PBS (pH 7.2), 50 μl of myoglobin solution, and 20 μl of 3 mM ABTS. Reaction was initiated with H_2_O_2_ (20 μl) and change in color monitored at 570 nm (standard curve: 2–25 μM trolox).

### Transient transfection and NFκB activity assay

The NFκB-TATA-luciferase reporter plasmid was graciously provided by Dr. Shao-Cong Sun (University of Texas MD Anderson Cancer Center). The pRL-TK vector containing *Renilla* luciferase (positive control) was purchased from Promega (Madison, WI). HEK-293 cells (CRL-1573, ATCC Manassas, VA) were cultured in Dulbecco's Modified Eagle Medium (DMEM) media in T75 flasks. Cells were seeded in 96-well tissue culture plates (2.5X10^4^ cells/well), allowed to adhere for 1 h, and acclimatized overnight to antibiotic-free Opti-MEM medium. Cells were transfected with NFκB-TATA-luciferase (100 ng) and pRL-TK (10 ng) using Lipofectamine 2000 (Invitrogen) according to the instructions provided by the manufacturer. After 6 h of incubation, cells were washed, replenished with complete medium for 3–5 h, and then infected with *T*. *cruzi* (cell: parasite ratio, 1:3). Cells were incubated in presence or absence of 1 μM each of SRT1720 for 24 h. The relative NFκB transcriptional activity was measured by using a Dual Luciferase Reporter Assay System (Promega, Madison, WI) and data were normalized to *Renilla* luciferase activity.

### Data analysis

All experiments were conducted with triplicate observations per sample (n = 6 mice per group per experiment, at least two experiments per group), and data are expressed as mean ± standard deviation (SD). All data were analyzed using GraphPad Prism 5 (GraphPad Software, La Jolla, CA). Data were analyzed by Student’s t test (comparison of 2 groups) and one-way ANOVA with Tukey’s test (comparison of multiple groups). Significance is presented by * (infected vs. normal) or ^#^ (infected/treated vs. infected/untreated) (*^, #^p<0.05, **^,##^p<0.01, ***^,###^p<0.001).

## Supporting Information

S1 Table List of oligonucleotides used in this study (DOCX)

S1 Fig Resveratrol was partially beneficial in controlling the left ventricular (LV) dysfunction in chagasic mice. C57BL/6 mice were infected with *Trypanosoma cruzi* (10,000 *Tc*/mouse) and treated orally with resveratrol (20 mg/ml) during 90–111 days post-infection (pi). Transthoracic echocardiography was performed at ~150 days pi using a Vevo 2100 System. Shown are bar graphs for end systolic volume (panel a), stroke volume (SV, panel b), cardiac output (CO, panel c), ejection fraction (EF, panel d), left ventricular internal diameter at systole (LVIDs, panel e), and fractional shortening (FS, panel f). In all figures, data are presented as mean value ± SD (n = 6–10 mice per group per experiment). Significance was calculated by one-way ANOVA with Tukey’s test and plotted as *^,#^p<0.05, **^,##^p<0.01, ***^,###^p<0.001 (*normal control vs. *Tc*-infected, ^#^ *Tc*-infected vs. *Tc*-infected/resveratrol-treated).
(TIF)

S2 Fig Effects of resveratrol on cardiac remodeling in chagasic mice. Mice were infected, resveratrol-treated, and monitored as in S1 Fig. Bar graphs show interventricular septum thickness at systole (panel a) and diastole (panel b), LV posterior wall thickness at systole (panel c), LV mass (panel d) and LV area at systole (panel e). Significance was calculated and presented as in S1 Fig.
(TIF)

S3 Fig Effect of resveratrol on mitochondrial biogenesis in chagasic mice. C57BL/6 mice were infected with *T*. *cruzi*, treated with resveratrol, and harvested at ~150 days pi. **(A)** Myocardial mtDNA content by qPCR for (a) CYTB and (b) COII regions of mtDNA was normalized to β-globin nuDNA. **(B)** Quantitative RT-PCR for mtDNA encoded transcripts (ND1, COIII, ATP6, a-c panels) and mtDNA replication/transcriptional machinery (POLG1, SSBP1, TOP1, d-f panels). For each target gene, *C*t values were normalized to GAPDH expression. Significance was calculated and presented as in S1 Fig.
(TIF)

S4 Fig Oxidant/antioxidant status in chagasic mice (± resveratrol). Mice were infected with *T*. *cruzi*, treated with resveratrol, and heart tissues harvested at 150 days pi. Shown in panel a are representative immunoblots for inducible nitric oxide synthase (iNOS), 4-hydroxynonenal (4HNE), 3-nitrotyrosine (3NT), Nrf2, and GAPDH. Densitometry analysis of the western blot bands, normalized to GAPDH, is shown in panel b. Significance was calculated and presented as in S1 Fig.
(TIF)

S5 Fig Cytokine gene expression in chagasic mice (± resveratrol). Mice were infected with *T*. *cruzi* and treated with resveratrol. Myocardial expression level of (a) IFNγ, (b) IL1β, and (c) TNFα mRNAs was determined at 150 days pi by qRT-PCR. For each target gene, *C*t values were normalized to GAPDH expression. Significance was calculated and presented as in S1 Fig.
(TIF)
