# Supplementary material for: SIRT1-PGC1α-NFκB Pathway of Oxidative and Inflammatory Stress during Trypanosoma cruzi Infection: Benefits of SIRT1-Targeted Therapy in Improving Heart Function in Chagas Disease
Source: PLoS Pathog. 2016 Oct 20;12(10):e1005954. doi: 10.1371/journal.ppat.1005954 (PMC5072651; doi:10.1371/journal.ppat.1005954)
Supplement: S1 Table — (DOCX) [file ppat.1005954.s001.docx]

| **S1 Table. Oligonucleotides used in this study** | | | | | | | |
| --- | --- | --- | --- | --- | --- | --- | --- |
| **Gene Name** | **Protein**  **Name** | **Genbank Accession #** | | **Oligo-nucleotide** | **Oligonucleotide**  **sequence 5'-3'** | **Amplicon size (bp)** |  |
| **mRNA amplification** | | | | |  |  |  |
| ND1 | NADH dehydrogenase subunit 1, mt | [NC_001569](http://www.ncbi.nlm.nih.gov/entrez/query.fcgi?cmd=Search&db=Nucleotide&term=NC_001569) | | ND1 F | TCACTATTCGGAGCTTTACGAGC | 173 |  |
|  |  |  | | ND1 R | CATATTATGGCTATGGGTCAGGC |  |  |
| COIII | Cytochrome oxidase complex subunit III, mt | [NC_005089.1](http://www.ncbi.nlm.nih.gov/entrez/viewer.fcgi?db=nucleotide&id=34538597) | | COIII F | TGCTGACCTCCAACAGGAAT | 198 |  |
|  |  |  | | COIII R | TTCTGAAGCTTGGAGGATGG |  |  |
| ATP6 | ATP synthase 6, mt | [NC_000067.6](http://www.ncbi.nlm.nih.gov/nucleotide/372099109?from=24614164&to=24614282&report=gbwithparts) | | ATP6 F | TCACTTGCCCACTTCCTTCC | 119 |  |
|  |  |  | | ATP6 R | TTAGCTGTAAGCCGGACTGC |  |  |
| POLG1 | DNA polymerase gamma subunit 1, mt | [NC_000073.6](http://www.ncbi.nlm.nih.gov/nucleotide/372099103?from=79461877&to=79464825&report=gbwithparts) | | POLG1 F | GAGCCTGCCTTACTTGGAGG | 294 |  |
|  |  |  | | POLG1 R | GGCTGCACCAGGAATACCA |  |  |
| SSBP1 | Single-stranded dna binding protein, mt | NC_000072.6 | | SSBP1 F | CAACAAATGAGATGTGGCGATCA | 565 |  |
|  |  |  | | SSBP1 R | ACGAGCTTCTTACCAGCTATGA |  |  |
| TOP1 | Topoisomerase (DNA) I, mt | XM_005392521.2 | | TOP1 F | GACCATCTCCACAACGATTCC | 96 |  |
|  |  |  | | TOP1 R | ATGCCGGTGTTCTCGATCTTT |  |  |
| COLI | Collagen I | [NC_000077.6](http://www.ncbi.nlm.nih.gov/nucleotide/372099099?from=94950704&to=94950861&report=gbwithparts) | | COLI F | GAGCGGAGAGTACTGGATCG | 158 |  |
|  |  |  | | COLI R | GCTTCTTTTCCTTGGGGTTC |  |  |
| COLIII | Collagen III | [NC_000067.6](http://www.ncbi.nlm.nih.gov/nucleotide/372099109?from=45344469&to=45345003&report=gbwithparts) | | COLIII F | GTCCACGAGGTGACA AAGGT | 535 |  |
|  |  |  | | COLIII R | GATGCCCACTTGTTCCATCT |  |  |
| αSMA | Smooth muscle actin alpha | [NC_000080.6](http://www.ncbi.nlm.nih.gov/nucleotide/372099096?from=54957801&to=54958669&report=gbwithparts) | | SMA F | CTGGGCAAATCCAACAACTT | 869 |  |
|  |  |  | | SMA R | TCTTGCCTCCTTTGCCTTTA |  |  |
| IFNγ | Interferon-gamma | [NC_000076.6](http://www.ncbi.nlm.nih.gov/nucleotide/372099100?from=118441232&to=118442779&report=gbwithparts) | | IFNG F | CATTGAAAGCCTAGAAAGTCTG | 201 |  |
|  |  |  | | IFNG R | CTCATGAATGCATCCTTTTTCG |  |  |
| IL1β | Interleukin-1 beta | [NC_000068.7](http://www.ncbi.nlm.nih.gov/nucleotide/372099108?from=129367326&to=129367400&report=gbwithparts) | | IL1B F | GAGCTTCAGGCAGGCAG | 459 |  |
|  |  |  | | IL1B R | GGGATCCACACTCTCCAGC |  |  |
| IL10 | Interleukin 10 | [NM_010548.2](http://www.ncbi.nlm.nih.gov/entrez/viewer.fcgi?db=nucleotide&id=291575143) | | IL10 F | GCTCTTACTGACTGGCATGAG | 103 |  |
|  |  |  |  | IL10R | CGCAGCTAGGAGCATGTG |  |  |
| Arg1 | Arginase 1 | [NM_007482.3](http://www.ncbi.nlm.nih.gov/entrez/viewer.fcgi?db=nucleotide&id=158966684) | | Arg1 F | CAGAAGAATGGAAGAGTCAG | 249 |  |
|  |  |  |  | Arg1 R | CAGATAGCAGGGAGTCACC |  |  |
| TNFα | Tumor necrosis factor-alpha | [NC_000083.6](http://www.ncbi.nlm.nih.gov/nucleotide/372099093?from=35200275&to=35201110&report=gbwithparts) | | TNFA F | GTTCTATGGCCCAGACCCTCACA | 836 |  |
|  |  |  | | TNFA R | TACCAGGGTTTGAGCTCAGC |  |  |
| GAPDH | Glyceraldehyde 3-phosphate | [NC_000067.6](http://www.ncbi.nlm.nih.gov/nucleotide/372099109?from=12565651&to=186961180&report=gbwithparts) | | GAPDH F | TGGCAAAGTGGAGATTGTTG | 402 |  |
|  |  |  | | GAPDH R | TTCAGCTCTGGGATGACCTT |  |  |
| hIL1β | Interleukin 1 beta | [XM_017003988.1](http://www.ncbi.nlm.nih.gov/entrez/viewer.fcgi?db=nucleotide&id=1034613650) | | \| hIL1B F \| \| --- \| \| IL1B R \| | ACAGATGAAGTGCTCCTTCCA | 73 |  |
|  |  |  | | \| hIL1B F \| \| --- \| \| IL1B R \| | GTCGGAGATTCGTAGCTGGAT |  |  |
| hIL6 | Interleukin 6 | [XM_011515390.2](http://www.ncbi.nlm.nih.gov/entrez/viewer.fcgi?db=nucleotide&id=1034655490) | | \| hIL6 F \| \| --- \| \| IL10R \| | GTAGCCGCCCCACACAGA | 101 |  |
|  |  |  | | \| hIL6 F \| \| --- \| \| IL10R \| | CATGTCTCCTTTCTCAGGGCTG |  |  |
| **DNA amplification** | | | | |  |  |  |
| COII mtDNA | Cytochrome oxidase complex subunit II, mt | NC_012387 | | COII F | ATTGCCCTCCCCTCTCTACGCA | 402 |  |
|  |  |  | | COII R | CGTAGCTTCAGTATCATTGGTGCCC |  |  |
| CYTB mtDNA | Cytochrome b, mt | [NC_010339.1](http://www.ncbi.nlm.nih.gov/entrez/viewer.fcgi?db=nucleotide&id=167716836) | | CYTB F | GCAACCTTGACCCGATTCTTCGC | 71 |  |
|  |  |  | | CYTB R | TGAACGATTGCTAGGGCCGCG |  |  |
| β-Globin nuDNA | Beta globin | NC_000073.6 | | β-Glob F | AGCCACAGATCCTATTGCCATGC | 239 |  |
|  |  |  | | β-Glob R | TGTTGCTTGGTAAACACAGA |  |  |
| Tc18S rDNA | *T. cruzi* 18S ribosomal DNA | [NC_018331.1](http://www.ncbi.nlm.nih.gov/nucleotide/401424696?from=982402&to=991477&report=gbwithparts) | | *Tc*18S F | TTTT GGGC AACA GCAG GTCT | 200 |  |
|  |  |  | *Tc*18S R | | CTGC GCCT ACGA GACA TTCC |  |  |
| Unless specified, all oligonucleotides are based on mouse cDNA sequence. Human cDNAs are presented as hIL1β and hIL6. were | | | | | | |  |
