# Supplementary figures and images for: SIRT1-PGC1α-NFκB Pathway of Oxidative and Inflammatory Stress during Trypanosoma cruzi Infection: Benefits of SIRT1-Targeted Therapy in Improving Heart Function in Chagas Disease
Source: PLoS Pathog. 2016 Oct 20;12(10):e1005954. doi: 10.1371/journal.ppat.1005954 (PMC5072651; doi:10.1371/journal.ppat.1005954)

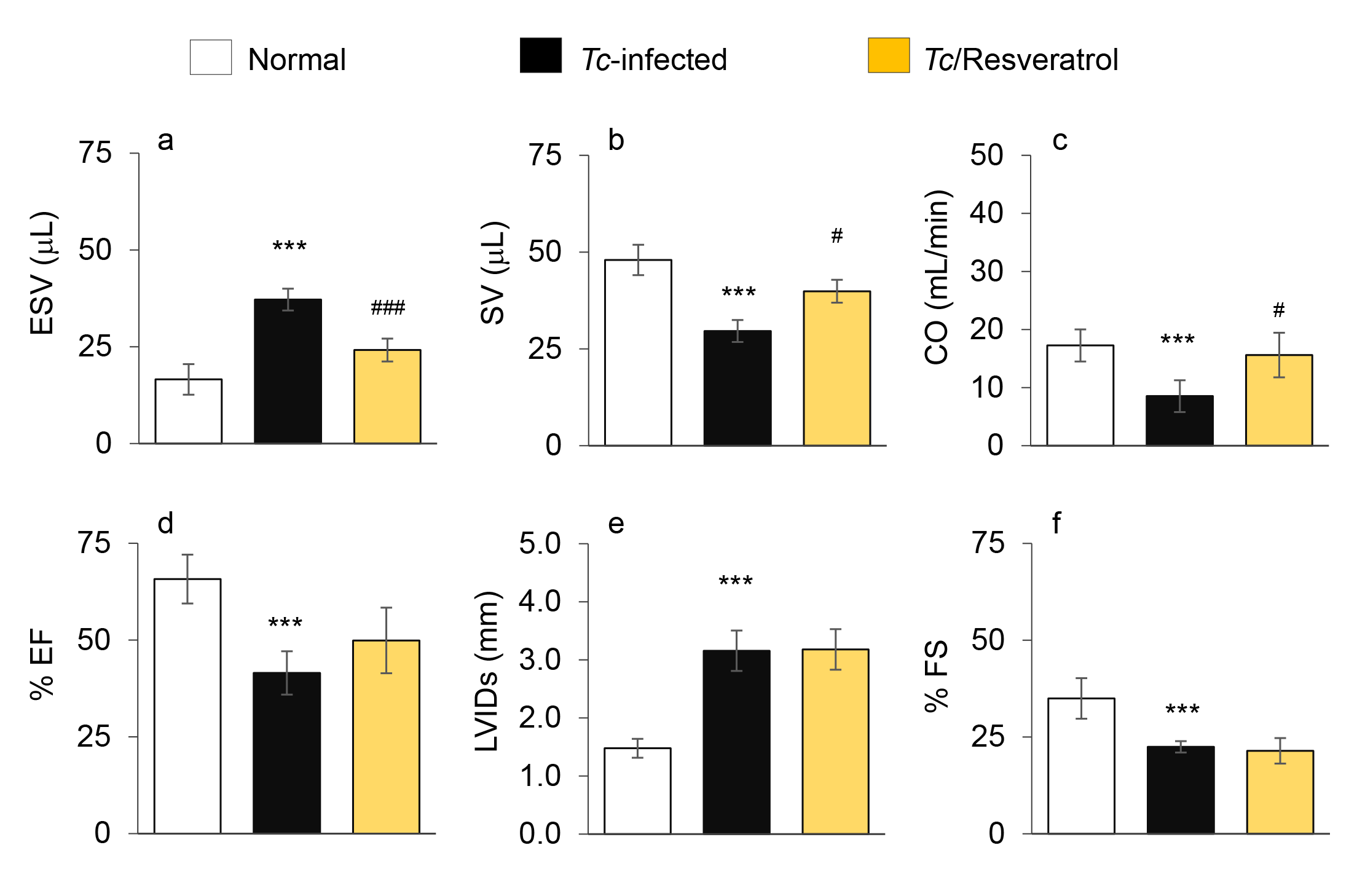

Supplement: S1 Fig — C57BL/6 mice were infected with Trypanosoma cruzi (10,000 Tc/mouse) and treated orally with resveratrol (20 mg/ml) during 90–111 days post-infection (pi). Transthoracic echocardiography was performed at ~150 days pi using a Vevo 2100 System. Shown are bar graphs for end systolic volume (panel a), stroke volume (SV, panel b), cardiac output (CO, panel c), ejection fraction (EF, panel d), left ventricular internal diameter at systole (LVIDs, panel e), and fractional shortening (FS, panel f). In all figures, data are presented as mean value ± SD (n = 6–10 mice per group per experiment). Significance was calculated by one-way ANOVA with Tukey’s test and plotted as *,#p<0.05, **,##p<0.01, ***,###p<0.001 (*normal control vs. Tc-infected, # Tc-infected vs. Tc-infected/resveratrol-treated). (TIF) [file ppat.1005954.s002.tif]

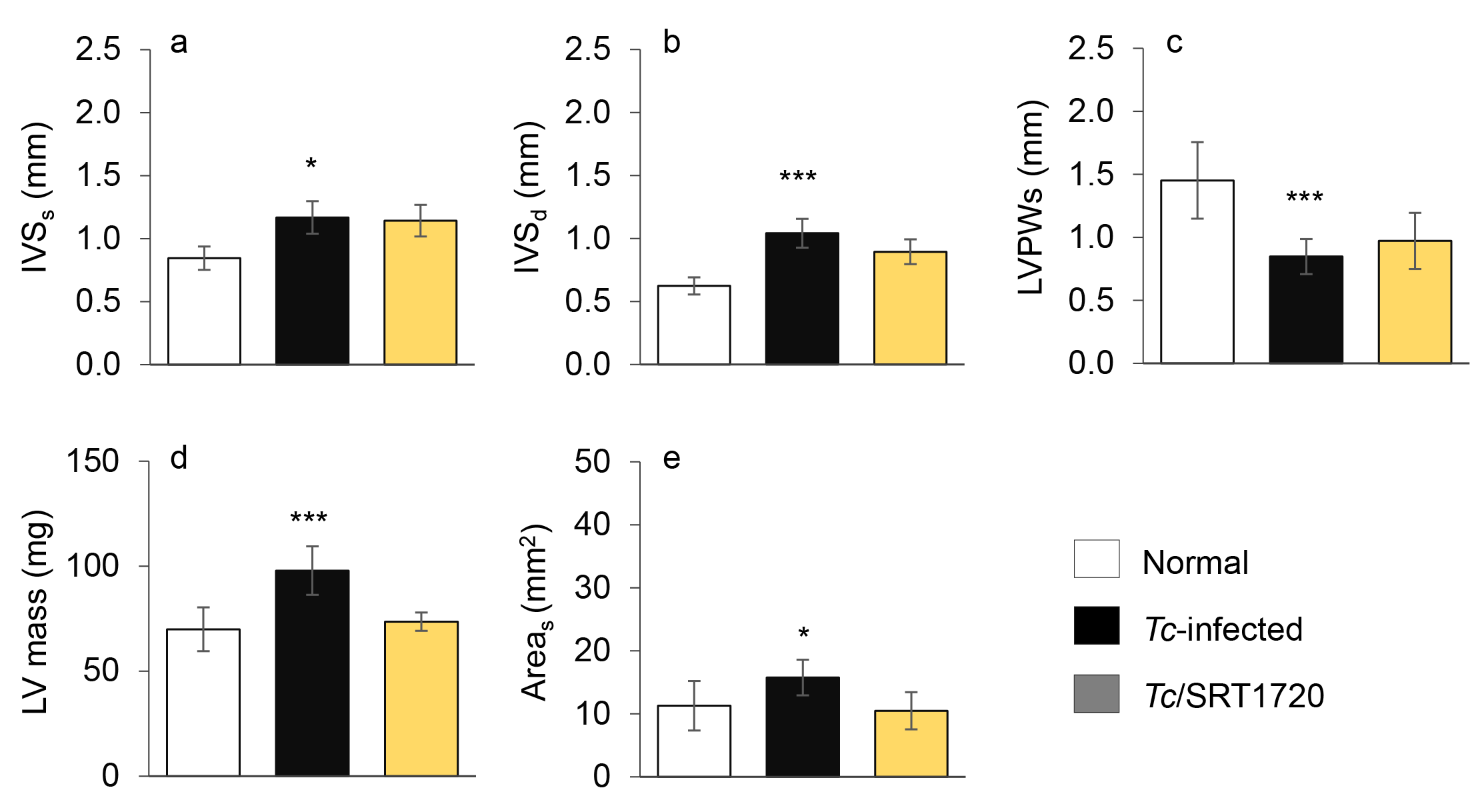

Supplement: S2 Fig — Mice were infected, resveratrol-treated, and monitored as in S1 Fig. Bar graphs show interventricular septum thickness at systole (panel a) and diastole (panel b), LV posterior wall thickness at systole (panel c), LV mass (panel d) and LV area at systole (panel e). Significance was calculated and presented as in S1 Fig. (TIF) [file ppat.1005954.s003.tif]

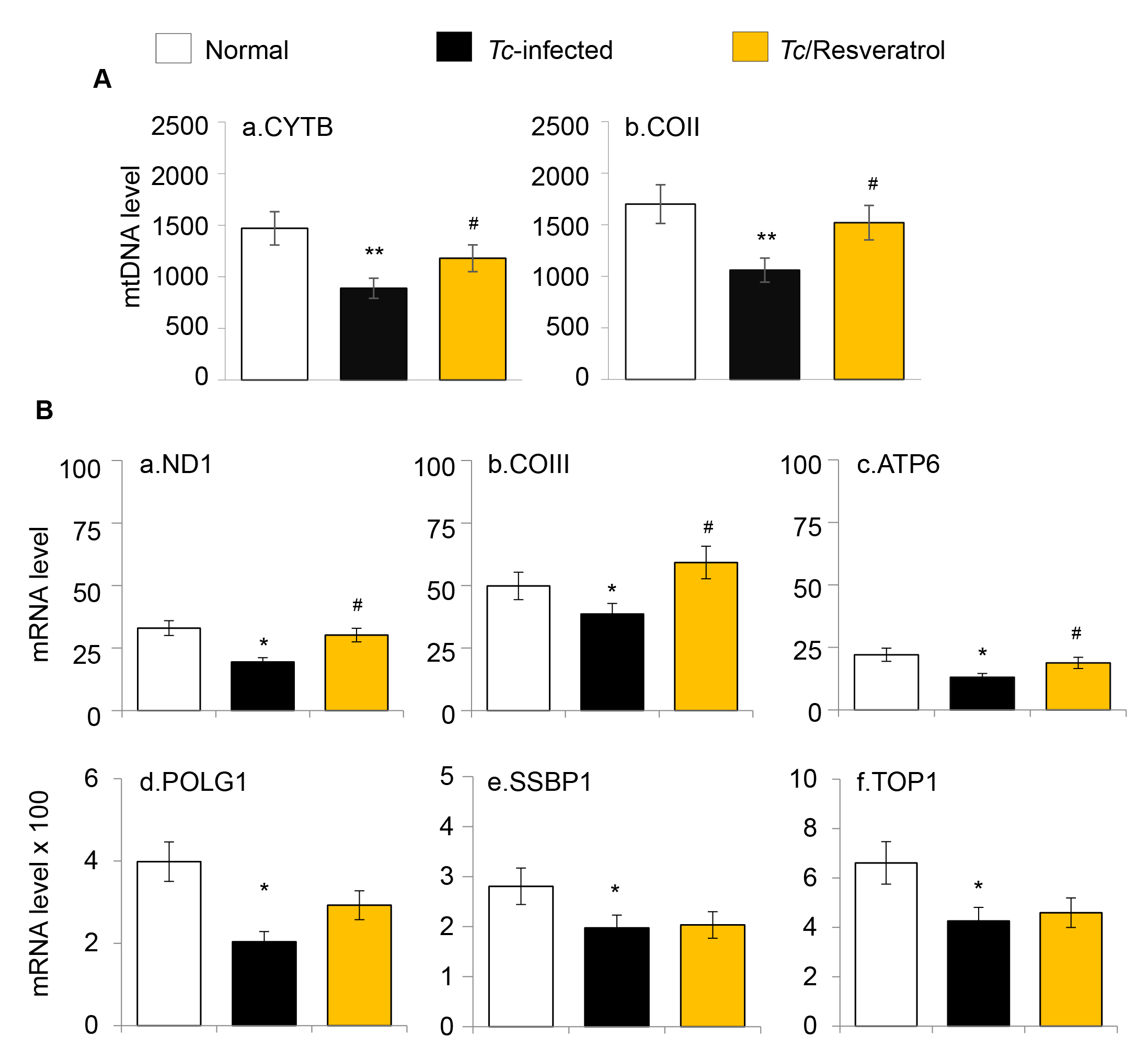

Supplement: S3 Fig — C57BL/6 mice were infected with T. cruzi, treated with resveratrol, and harvested at ~150 days pi. (A) Myocardial mtDNA content by qPCR for (a) CYTB and (b) COII regions of mtDNA was normalized to β-globin nuDNA. (B) Quantitative RT-PCR for mtDNA encoded transcripts (ND1, COIII, ATP6, a-c panels) and mtDNA replication/transcriptional machinery (POLG1, SSBP1, TOP1, d-f panels). For each target gene, Ct values were normalized to GAPDH expression. Significance was calculated and presented as in S1 Fig. (TIF) [file ppat.1005954.s004.tif]

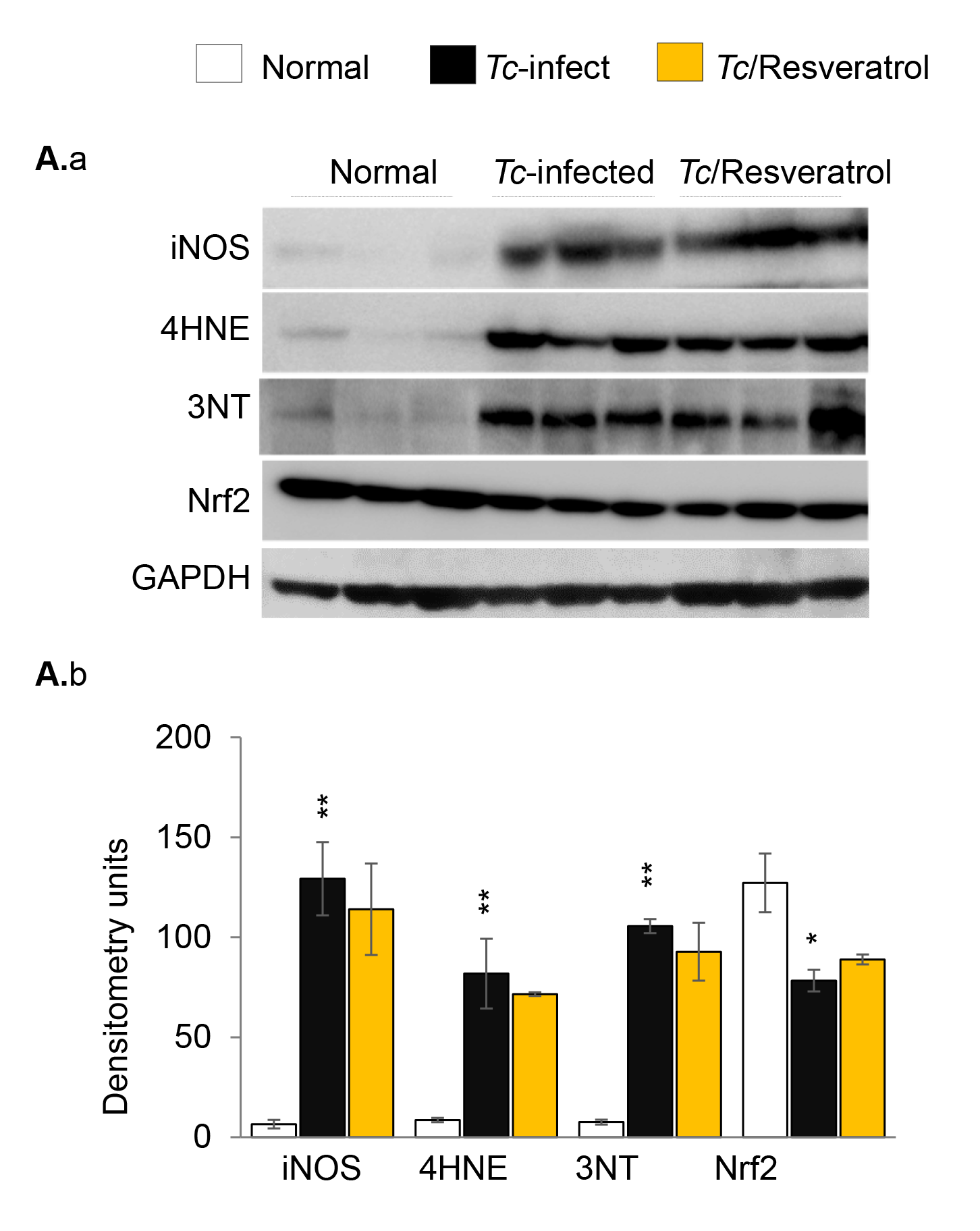

Supplement: S4 Fig — Mice were infected with T. cruzi, treated with resveratrol, and heart tissues harvested at 150 days pi. Shown in panel a are representative immunoblots for inducible nitric oxide synthase (iNOS), 4-hydroxynonenal (4HNE), 3-nitrotyrosine (3NT), Nrf2, and GAPDH. Densitometry analysis of the western blot bands, normalized to GAPDH, is shown in panel b. Significance was calculated and presented as in S1 Fig. (TIF) [file ppat.1005954.s005.tif]

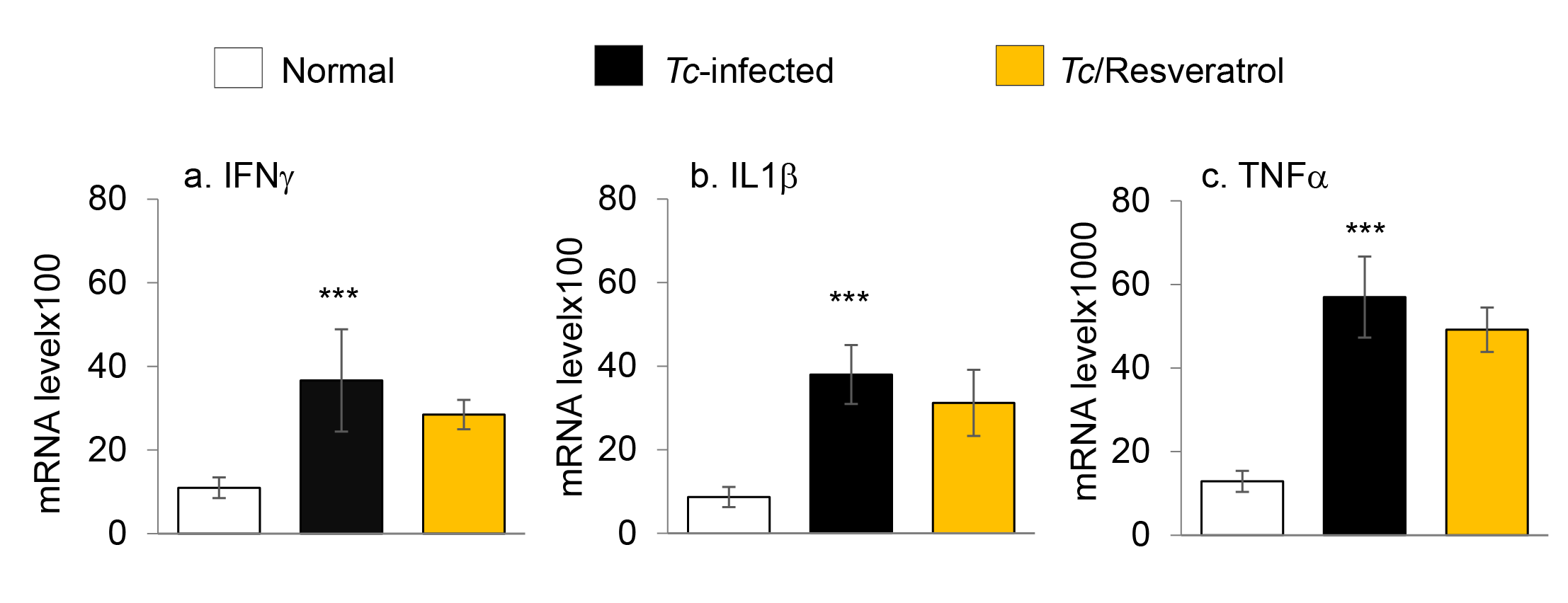

Supplement: S5 Fig — Mice were infected with T. cruzi and treated with resveratrol. Myocardial expression level of (a) IFNγ, (b) IL1β, and (c) TNFα mRNAs was determined at 150 days pi by qRT-PCR. For each target gene, Ct values were normalized to GAPDH expression. Significance was calculated and presented as in S1 Fig. (TIF) [file ppat.1005954.s006.tif]
